# Flexible Packaging by Film-Assisted Molding for Microintegration of Inertia Sensors

**DOI:** 10.3390/s17071511

**Published:** 2017-06-27

**Authors:** Daniel Hera, Armin Berndt, Thomas Günther, Stephan Schmiel, Christine Harendt, André Zimmermann

**Affiliations:** 1 Institute for Micro Assembly Technology, Hahn-Schickard e. V, Allmandring 9B, 70569 Stuttgart, Germany; Thomas.Guenther@Hahn-Schickard.de (T.G.); zimmermann@ifm.uni-stuttgart.de (A.Z.); 2 Institut für Mikroelektronik Stuttgart, Allmandring 30a, 70569 Stuttgart, Germany; Berndt@ims-chips.de (A.B.); schmiel@ims-chips.de (S.S.); harendt@ims-chips.de (C.H.); 3 Institute for Micro Integration (IFM), University of Stuttgart, Allmandring 9B, 70569 Stuttgart, Germany

**Keywords:** packaging, MEMS, EMC, FAM, button shear test

## Abstract

Packaging represents an important part in the microintegration of sensors based on microelectromechanical system (MEMS). Besides miniaturization and integration density, functionality and reliability in combination with flexibility in packaging design at moderate costs and consequently high-mix, low-volume production are the main requirements for future solutions in packaging. This study investigates possibilities employing printed circuit board (PCB-)based assemblies to provide high flexibility for circuit designs together with film-assisted transfer molding (FAM) to package sensors. The feasibility of FAM in combination with PCB and MEMS as a packaging technology for highly sensitive inertia sensors is being demonstrated. The results prove the technology to be a viable method for damage-free packaging of stress- and pressure-sensitive MEMS.

## 1. Introduction

The production of smart packaging solutions presents a major challenge in microassembly technology [1,2]. Polymeric packages are commonly based on thermosets such as epoxy molding compounds (EMC), which are processed using transfer molding technology. EMCs offer a large scale of advantages like good chemical resistance, a high Young‘s modulus, very low coefficients of thermal expansion (CTE), decent thermal conductivity, etc. Even highly sensitive electronic assemblies employing chip-and-wire techniques can be packaged due to the reduced shrinkage and low viscosity of EMC compared to thermoplastic materials [3,4]. However, while transfer molding is the most commonly used technology for microsystem packaging [5], the technology is mainly offered in the Far East [6] and applied by packaging companies, which are interested in manufacturing large quantities and standard packages, such as small outline (SOP), pin grid array (PGA), quad flat no leads (QFN), land grid array (LGA), or ball grid array package (BGA) [7,8]. Since small or medium sized companies in Europe often require small production volumes with individual packaging solutions for their highly innovative microsystem packages, standard production in the Far East is often a non-viable alternative.

Film assisted transfer molding (FAM) is based on standard transfer molding (TM) technology and presents a new, advanced industrial manufacturing technology [9]. The major modification includes the application of a foil, mostly made of ethylen-tetrafluoroethylene (ETFE), which presents a perfect protection of the tool from the EMC besides its main purpose as a non-adhesive layer. The foil, which is replaced with each cycle, leads to a virtually unlimited tool-life and therefore to a ‘first part identical to last part’ principle. Furthermore, abrasion and tool complexity is reduced, as there is no requirement for mechanical ejector pins. Additionally, the foil seals the tool, so that tool-making is simplified with regard to precision and tolerances. However, the major feature of the foil is the possibility that the tool can touch and seal sensitive sensor surfaces without causing damages.

MEMS are typically packaged in hard shell ceramic or metal encapsulation because of the mechanical component of the sensor, which could become dysfunctional after packaging by an encapsulation technology with direct contact to the sensor elements. Thus the cost for producing such a MEMS package is commonly high due to the complexity of the encapsulation and its assembly steps. Combining the PCB and FAM technology, low cost MEMS packaging is possible. With the developments of the IGF (Industrielle Gemeinschaftsforschung) project ‘FlexPacFAM’, founded by the German state [10], a new set of packages becomes available. These packages, labeled as FlexPacs, use the high design flexibility of PCB technology for precise and aligned MEMS packaging.

The advantage of PCBs in comparison to lead frames is that they can be easily designed using available software tools and produced in a cost-efficient manner by employing multilayer technology for complex routed electrical circuits. Thereby, a much higher integration density and complexity can be achieved in comparison to lead frames. This allows the integration of a smart system in the package. In particular, leadless packages like QFN are in high demand due to the trend to use less space for an assembly. Providing an even further miniaturization, more complex circuitry can be embedded into the QFN [11]. 

On the downside of the combinatory technology, PCBs show higher tolerances than lead frames. For this reason, alignment methods for FAM processes are not trivial [1]. The challenges in combining FAM, PCB and MEMS arise in particular from differing tolerance levels of each technology. Dimensional accuracy of PCB technology compared to MEMS is significantly lower. On the one side, this requires that the packaging process is able to handle highly precise MEMS, which are sensitive to mechanical stresses caused by misaligned configurations; on the other side, a process which can handle the large deviations in geometrical dimension of the PCB is required. 

With the new developments, the FAM process, in combination with MEMS and PCB, was enabled to be employed for scalable, flexible production and low-cost MEMS sensor encapsulation, whereas the requirements of MEMS remained unknown regarding a damage-free encapsulation. 

In order to evaluate the combination of technologies regarding its reliability for highly sensitive sensors, the electrical measurement signal of MEMS was analyzed after encapsulation with EMC, proving the applicability of this new technology for flexible, functional packages.

## 2. Materials and Methods 

For the FAM process, a Unistar Boschman FAM machine with the ability to process EMC pellets was used for the development of the flexible, PCB-based packaging concept. The material EME-G770H Type CD from Sumitomo Bakelite was selected in order to provide a good CTE match for die packaging on PCB. The process temperature was set to 180 °C, at which the ETFE foil remained stable during the FAM process while becoming flexible for a form adaptation into the mold cavity. At the end of the FAM process cycle, the used foil was coiled onto a roll for disposal, and at the same time new foil was placed into the process area.

Due to the properties of the PCB technology, the adhesion mechanisms of EMC on the PCB surface are of utmost importance in relation to the reliability of the molded system. Thus, adhesions tests were carried out with defined EMC buttons molded onto the substrate’s different surfaces and subsequent shear testing with a DAGE Series 4000. The tests were designed according to the SEMI standard G69-0996 [12]. The test panel was a PCB with three kinds of surfaces: PCB base material (FR4), solder resist and metal pads with ENEPIG gold finish. ENEPIG is a chemical copper (Cu) nickel (Ni) palladium (Pd) gold (Au) layer on the PCB, commonly used for bare die attachments by flip chip and wire bonding. The main part of this metal layer consists of Cu as a conductive layer, but the bonding surface itself is made of Au for reliable bonding with other gold systems. The glass transition temperature of the PCB was 150 °C and the base material was a halogen reduced flame-retardant 4 material (FR4). 

**Figure 1:**
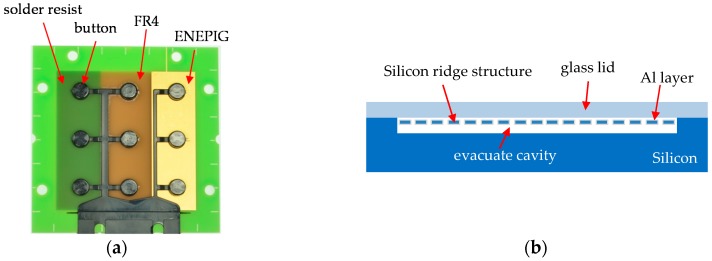
(**a**) Test vehicle for the button shear test to characterize the adhesion strength of the epoxy molding compounds (EMC) on solder resist, FR4 and metal pads with ENEPIG (from left) and (**b**) schemata of the inertia sensor construction.

An insert for the molding tool was designed and manufactured by precision tool making to mold the EMC buttons on the test panel by means of the FAM process (Figure 1a). For each kind of surface, three buttons were generated. A total of 18 EMC buttons were sheared from each type of surface in order to quantify the adhesion of the EMC on different PCB surfaces. Additionally, treatment of the surfaces by low pressure oxygen plasma (O_2_-plasma) and conventional atmospheric plasma (N_2_/O_2_-plasma) were also tested to characterize the influence of these methods regarding the adhesion properties.

For the investigation of packaging MEMS on PCB using FAM, an inertia sensor with a thin aluminum (Al) layer for the electrical conductivity of the silicon ridge structure was used. This type of sensor is typically used in applications such as construction machinery to measure inclination. The sensor is based on silicon technology (MEMS) and a glass lid acts as a protection mounted on top of the sensor to cover the MEMS structures (Figure 1b). Normally, these MEMS are packaged in a ceramic package to avoid mechanical pressure on the Al layer and to minimize internal stresses. For signal interpretation of the measured signal, an application-specific integrated circuit (ASIC) can be mounted on the glass lid and wire bonded down to the electrical contact pads of the inertia sensor as well as the carrier substrate.

The investigation was divided into two parts. Throughout the first part, the aim was to show how low the cure pressure within the FAM process needs to be to achieve complete, flawless molding without any damage of the inertia sensors. The cure pressure is the parameter, which takes the most stress onto the sensor. After the virtually pressure-free transfer of the EMC melt into the cavity by simple displacement of the air with material, both at atmospheric pressure, the cure pressure starts to compress the EMC and drives out the remaining air out of the cavity. Subsequently, the cure pressure values increase to 30 bar to 60 bar in typical applications, which can damage the Al layer by deforming the glass lid of the sensor and consequently the sensor itself. 

For the first approach, five PCB panels with nine sensors each were mounted. Each PCB was equipped with four partly malfunctioning inertia sensors, where the x-axis was working unobtrusively but the y-axis was malfunctioning. Five dummy sensors were bonded at the empty slots in order to provide thermal stability and material distribution equally to the final constellation in a later production cycle. 

After the transfer process of the EMC melt, four different cure pressures were applied, namely 10 bar, 20 bar, 30 bar and 40 bar. One of the PCB panels underwent the FAM process without encapsulation with EMC to verify the effect of process temperature on the inertia sensor. After FAM, an electrical test by means of CVC2 (capacity-to-voltage converter with two channels) circuit was carried out to measure the static and dynamic behavior of the packaged inertia sensor. The results were compared to evaluate the best cure pressure for production. Applying X-ray using computer tomography (HMXST 160, X-TEK Systems Ltd., Tring, Herts, UK), the cured EMC was inspected to analyze the density and identify voids within the material.

The second approach employed fully functional inertia sensors in a stack configuration with an ASIC mounted on the glass lid. Five PCB panels with five stacks were bonded on each panel. The sensor stack was programmed using the CVC2 circuit of the ASIC in order to get the sensor into a calibrated condition. The cure pressure of the FAM process was set to the smallest pressure that could be used to fill the whole cavity as evaluated in the first approach. An electrical measurement was carried out at four different stages: At first, after die and wire bonding and before the FAM process in order to record the electrical properties of each sensor after calibration. Secondly, after the FAM process to verify the signal changes caused by the encapsulation. Thirdly, after a post-mold cure process for EMC residual curing at 180 °C for 4 h. Finally, after a dicing process, which separates the inertia sensor-FlexPacs from the PCB panel. 

For each case, the electrical measurement was the same. The sensor capacity in *pF* in the x- and y-axis of the sensor was determined for an angle of 0° and 20°. Therefore, the offset capacity was measured to detect the influence of each process step by FAM.

To determine the warpage within the package due to internal stresses as a reason for increased offset capacity of the inertia sensor, a white light interferometry measurement from was carried out using a Veeco interferometer. 

## 3. Results and Discussion

### 3.1. Button Shear Test

**Figure 2:**
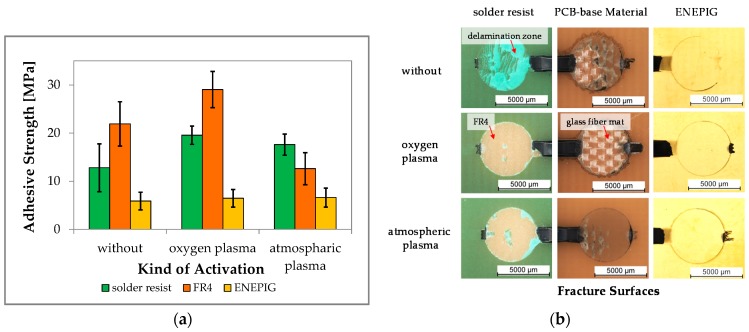
(**a**) The graphic shows adhesion strength in comparison to the kind of surface and activation. PCB-base materials demonstrate the highest adhesive strength in this investigation, and surface activation by means of oxygen plasma increases adhesive strength; (**b**) Presents the fracture surface of the different surfaces without and with plasma treatment. Polymeric surfaces exhibit cohesive fracture and metal surfaces show adhesive fracture.

The FR4 showed the strongest and the ENEPIG the lowest adhesive strength (Figure 2a). Treatment of the surfaces by O_2_-plasma significantly increased the adhesive strength effect on polymeric surfaces but it exerted only a weak effect on metallic ENEPIG surfaces. This stronger effect of oxygen plasma on polymeric surfaces was probably based on a better continuance of ionic oxygen on a polymeric surface compared to a metallic surface, leading to an improved wetting of the EMC on the polarized polymer surface. The Pd content of ENEPIG is typically a barrier for oxidation of Ni, preventing oxidation in order to improve the Au wire bonding on this surface. Solely a cleaning effect can be achieved using oxygen plasma on metal surfaces, whereas the ions react with organic contamination and evaporate. 

N_2_/O_2_-plasma treatment resulted in no significant change of the adhesive strength of EMC on ENEPIG. However, the PCB-base material showed a decreasing effect for the adhesion value in comparing to EMC on PCB without any pretreatment. This could be due to the fact that treatment of FR4 by means of N_2_/O_2_-plasma leads to a degeneration process of the FR4, whereas organic material evaporates out of the surface. Furthermore, some additives of the polymeric component may react with the nitrogen ions in the plasma, and the surface becomes more nonpolar in contrast. The effect of N_2_/O_2_-plasma treatment on solder resist showed an increased adhesion value, leading to the general conclusion that it is also possible to increase the adhesion values on polymeric surfaces.

By comparing both treatments, it could be recognized that the O_2_-plasma had a greater effect on increasing the adhesion on polymeric surfaces than the N_2_/O_2_-plasma treatment. For ENEPIG, no differences between the treatments could be observed.

Since the adhesion of EMC to FR4 without pretreatment provided sufficient strength (typ. > 5 MPa, rule of thumb), the process of plasma treatment is only recommended if increased reliability is required. Nonetheless, the plasma treatment presents also a method to remove surface contamination, thereby providing a safer process regarding the quality of differently produced PCBs. Furthermore, less statistical variations in terms of adhesion occur if plasma treatment is employed. 

Visual inspection of the fracture surfaces of each kind of surface showed cohesive failure for both solder resist and FR4. Adhesive fracture was observed for the ENEPIG surface (Figure 2b). Adhesion of EMC to solder resist was stronger than the adhesion of solder resist to FR4. This is detectable at the fracture surface of the solder resist with plasma treatment in Figure 2b, whereas the solder resist remained on the sheared EMC button and lifted off the FR4, leaving a circular area without any solder resist. At the non-plasma treated solder resist surfaces, delamination of the solder resist from the FR4 was observed, represented by the brighter-green surface areas. 

Since the fractured surface of the FR4 base material showing the glass fiber mat, which is located below the top surface of the PCB-base material, it can be concluded that the adhesion between EMC and the FR4 is higher than the adhesion between the different core layers of the PCB.

For the FR4 surfaces without plasma pretreatment, a cohesive fracture between EMC buttons to the FR4 was observed, indicated by the mixed adhesive and cohesive fracture between EMC button, FR4 and the glass fiber mat. The reason for the mixed adhesive/cohesive fracture might be that the EMC button lifts off the FR4 at the beginning of the shear process until a critical strength is reached. After exceeding a critical strength, the influence of tensile stress on FR4 epoxy generates a fracture path vertical to the shear direction that the EMC button pulls out the FR4 epoxy by braking from the surface. The atmospheric plasma reduces the adhesion of EMC on FR4 compared to non-treatment. In this case the fracture surface shows predominant an adhesive fracture. 

Consequently, solder resist, if possible, should be avoided if possible as it presents a weak point in terms of reliability. Thus, the following investigations were performed without solder resist on the top surface of the PCBs and without plasma pretreatment. However, if the area of exposed ENEPIG lines is significantly higher than FR4, solder resist on top of this surface is preferable to improve adhesion.

### 3.2. Investigation on Inertia Sensor: Maximum Allowed Cure Pressure

**Figure 3:**
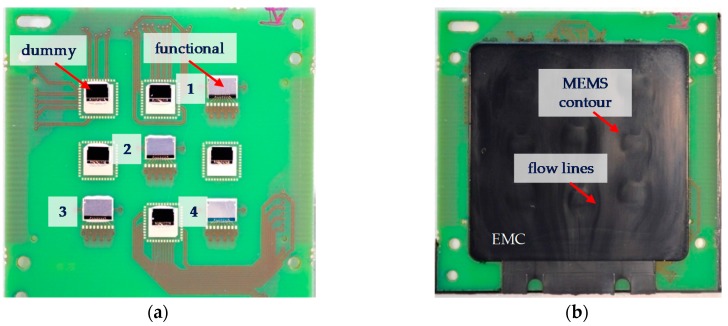
(**a**) Mounted inertia sensors on a PCB panel numbered from one to four and (**b**) representative image of an encapsulated panel after the film-assisted transfer molding (FAM) process.

Four partly functional inertia sensors were attached on three corners as well as in the middle of the PCB panel. In order to track the positions, the sensor positions were numbered from one to four (see Figure 3a). Empty slots were filled up by attaching dummies to provide a balanced thermal stability during FAM and cooling process. A total thickness of EMC of 1.3 mm was applied as encapsulation of the MEMS (Figure 3b). The contour of each element could be observed on the EMC surface. This results in a visual effect on the surface of the encapsulation, which is assumed to be a result of the differing separation of the release agent from the EMC during the filling process on top of the MEMS and PCB. Thereby, the surfaces become partially dull above the MEMS components. 

**Figure 4:**
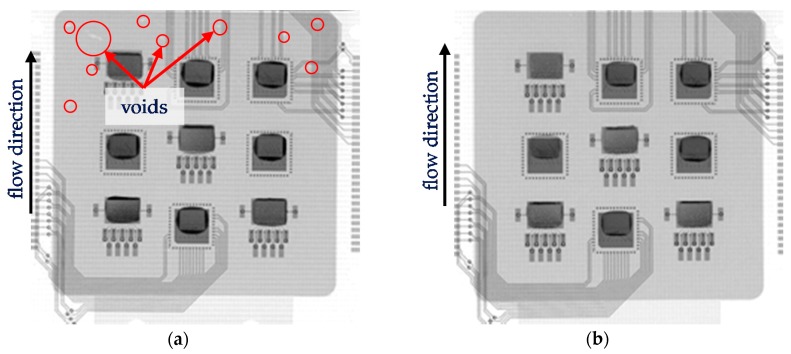
X-ray image from a PCB panel encapsulated using (**a**) 10 bar and (**b**) 30 bar cure pressure. Red circles marks voids.

Even though a common cure pressure above 20 bar is recommended for FAM, the visual inspection after FAM showed that all used cure pressure were able to fill the whole cavity. Therefore, 10 bar could be qualified for use as cure pressure for the main investigation. However, using X-ray, voids could be observed on the end of the cured flow front for cure pressure 10 bar (Figure 4a). That means that such a low cure pressure is not enough to achieve good density in the whole EMC. At 30 bar cure pressure, no voids were observable (Figure 4b). 

The results for the static electrical function test showed no discrepancies, meaning the electrical conductivity was checked successfully for each inertia sensor. However, the dynamic electrical test, at which the sensitivity of each inertia sensor was determined by an inclination of 30°*,* showed random results. All inertia sensors with a fail showed no or less sensitivity (Table 1).

The reference showed one fail and three functional inertia sensors. According to the manufacturer’s experience, such failures can result from die attachment processes, where handling can lead to damages of the glass lid and thus non-hermeticity, which in return causes the silicon ridge structure to lose its sensitivity. Also, thermal stresses based on the temperature cycle of the FAM process are a possible cause for the failure. Comparing the electrical test results, the test panel processed at a cure pressure of 30 bar had three functional inertia sensors after encapsulation. The other test panels showed more failed sensors after encapsulation. Hence, no connection between the cure pressure and the failure of the inertia sensors could be established, excluding the cure pressure as the main reason for failure. However, due to the complexity of the hybrid assembly of MEMS, die attach and FAM process, interactions of thermal and mechanical stresses cannot be excluded. Consequently, the dynamic electrical test demonstrates that by means of FAM, packaging with the tested curing pressures can be successful in terms of the MEMS sensors remaining functional. The yield needs to be evaluated and a detailed failure mode evaluation analysis with larger sample sets remains to be conducted.

**Figure 5:**
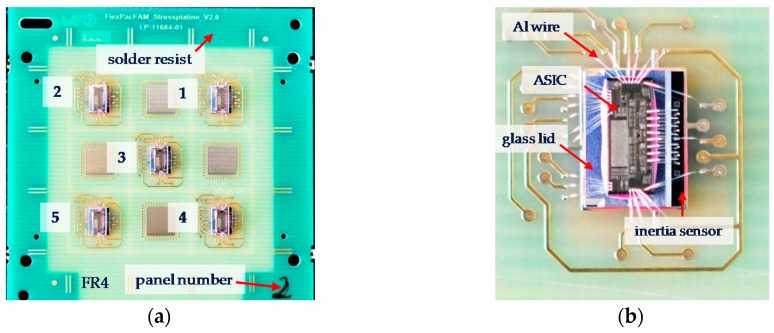
(**a**) Test panel with five mounted sensor stacks and (**b**) detailed view of the stack assembly of the inertia sensor and ASIC on PCB with aluminum (Al) bond wires as electric interconnect to die and PCB.

In the second investigation, five test panels were assembled with five fully functional inertia sensors, each with a stacked ASIC (Figure 5a). The wire bonding was done by means of Al wire bonding of the ASIC to the inertia sensor and the test panel (Figure 5b). Subsequent to assembly, the stacks were electrically tested to determine the electrical functionality and offset of each stack (Table 2). The x- and y-channel of each inertia sensor were detected for a 0° and 25° angle and the sensitivity of the capacity signal was determined. Mounted sensors, which showed no or too little sensitivity regarding specifications, were considered dysfunctional. For all other sensors, the offset capacity (hereinafter the “offset”) was determined. If the offset grade is 0, then the inertia sensor is in a calibrated electrical state. Increasing the offset value to ±1.5 pF means a degradation of the sensor signal due to stress on the glass lid. The stress causes an increased or decreased distance of the Al layer at the bottom of the glass lid to the silicon ridge. In the worst case, contact of the glass lid with the silicon ridge structure can cause destruction of the Al layer, leading to a failure of the sensor.

The first electrical tests after assembly and prior to encapsulation showed that three stacks were dysfunctional after the assembly process and one channel of stack at one of the panels was also dysfunctional. Some of the stacks had an offset signal, leading to reduced sensitivity or a signal that exceeds the calibrate range in maximum positions, but were tolerable according to the specifications. This offset is due to pre-damaging caused during the production of the MEMS or during the assembly procedure. 

### 3.3. Investigation on Inertia Sensor: Process Chain Optimization for An Inertia Sensor-FlexPac

Subsequent to a parameter screening for FAM process for a complete filling without voids, the set cure pressure could be minimized to 21 bar, thereby generating as little stress as possible to the sensor by the EMC melt. Subsequent electrical testing showed that for most of the stacks, the offset increased after encapsulation by the FAM process (Table 3). These sensors in particular showed a breakdown, which had an offset prior to molding. The sensor position 1 and 2 on the panel showed a larger variation of the offset after FAM compared to the other positions. This effect can be linked to pressure gradients during FAM, where the higher grade of cross-linking in the flow front cause more stress on the glass lid than on positions closer to the gate, which only are exposed to EMC melt where the grade of cross-linking is smaller. On the other hand, the effect of increasing the offset could also be caused by warpage of the test panel after FAM, which could be observed without any measurement tools. This warpage results from the mismatch of the coefficient of thermal expansion of each material in the hybrid package system, in particular the PCB and EMC.

**Figure 6:**
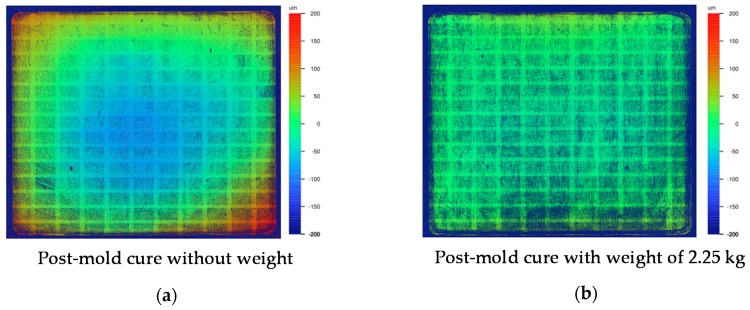
White light interferometry of the cured EMC surface after the post-mold cure of (**a**) test panel 2 without using a weight and (**b**) test panel 5 with using a weight of 2.25 kg.

After the post-mold cure, the warpage of test panels 2 and 5 was determined by means of white light interferometry (Figure 6). The difference between both test panels was that test panel 5 was applied with a weight of 2.25 kg on top during the post-mold cure process in order to compensate the warpage and flatten the substrate by influencing the stress during relaxation and cooldown. The warpage of panel 2 after the post-mold cure showed a concave warping of about 220 µm (maximum to minimum height). Applying the weight, the warping could be reduced to 40 µm. Notably, it was observed that after a reheat without weight, the warpage recovered, leading to the conclusion that the warpage compensation comprises residual stresses in polymeric chains and the interface between PCB and EMC. 

Electrical testing was performed after a post-mold cure, presented in Table 4. On test panel 1, the malfunctioning stacks recovered from dysfunctional and some channels were detected to work again. Conclusively, the post-mold cure presents a relaxation process of the EMC and stresses on the glass lid are reduced. Also, improvements in the offset values could be recognized, indicating that post-mold cure has a stress relaxation effect.

**Figure 7:**
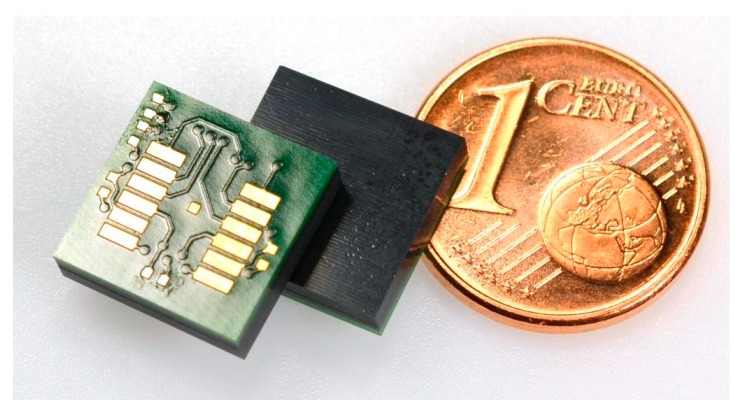
Separated inertia sensor-FlexPac.

Test panels 2 and 5 were diced using a wafer saw to separate the inertia sensor-FlexPacs in order to determine the influence of the dicing process on the stacks (Figure 7). The dicing process proved to have a mostly positive effect on the offset of panel 2 (Table 5). The warpage influence was reduced because of the smaller size of the FlexPac. The inertia sensor-FlexPac of panel 5 of position 3 showed a malfunctioning y-channel after dicing, for which vibration during dicing could be responsible. 

The results demonstrate that by means of FAM, inertia sensors on PCB can be functionally packaged. Furthermore, the stress on the MEMS can be reduced by a post-mold cure and dicing to separate the package from the panel. 

## 4. Conclusions 

This investigation successfully demonstrated that a PCB-based inertia sensor packaging solution can be realized by means of the film-assisted transfer molding technology. A button shear test for PCB was described, and two kinds of plasma were examined to determine design rules for the PCB and to quantify the best surface composition and pre-treatments. The adhesion of EMC on polymeric surfaces showed excellent adhesion strength and ENEPIG as the gold finish was in adhesion of EMC unaffected by plasma treatment. Furthermore, it was discussed that FAM is a functional solution to encapsulate pressure- and stress-sensitive MEMS as demonstrated by an inertia sensor-FlexPac. The process chain to assemble and package the inertia sensor-FlexPac was shown and validated by electrical function testing to determine the influence of each step. With cure pressure values above 10 bar in order to achieve void-free packaging, a certain degree of damage to the pressure-sensitive MEMS sensors was expected. However, it was recognized that mechanical, electrical and thermal failures are the dominant cause for malfunctions in the inertia sensor, not the cure pressure.

## Figures and Tables

**Table 1:** Results of the dynamic electrical test after FAM process of five test panels. The reference test panel underwent the FAM process without EMC encapsulation in order to be exposed to the same temperature record like the other four normally processed test panels.

Test Panel	10 bar	20 bar	30 bar	40 bar	Reference
1	ok	fail	fail	ok	ok
2	fail	fail	ok	ok	ok
3	ok	ok	ok	fail	ok
4	fail	fail	ok	fail	fail

**Table 2:** Results of the first electrical test of the five test panels after assembly process and prior to encapsulation. Green stands for a calibrated signal and orange for a signal within the tolerance range. Gray marks the dysfunctional channels.

Channel	Offset Panel 1		Offset Panel 2		Offset Panel 3		Offset Panel 4		Offset Panel 5	
	x	y	x	y	x	y	x	y	x	y
1	−0.1		−0.1	0	−0.1	0.1	−0.1	0	−0.1	0
2	1.2	1.5	0	0	−1.2	0	0	0	0	0
3	−1.2	1.3	0	0	0	0.1	0	0	−0.1	0.1
4			0	0			0.6	0	0	0
5	0	0	0	0	0	0			0	0

**Table 3:** Results of the second electrical test after encapsulation by FAM processing of the five test panels. Green stands for a calibrated signal and orange for a signal within tolerance range. Gray marks the channels that were dysfunctional from the beginning, and red shows malfunctioning stacks after encapsulation. The * identifies a change of the signal to the test before.

Channel	Offset Panel 1		Offset Panel 2		Offset Panel 3		Offset Panel 4		Offset Panel 5	
	x	y	x	y	x	y	x	y	x	y
1	1.5 *		−1.1 *	0	0 *	−1.2 *	0.2 *	1.2 *	0.3 *	1.2 *
2	1.2 *	1.5	−1.0 *	0.3 *	−1.2	0	0.1 *	0.1 *	0.2 *	0.1 *
3	−1.2	1.3	0.2 *	0.1 *	0.3 *	0.1	0.2 *	0	0.3 *	1.5 *
4			0.2 *	0			−1.5 *	0	−0.2 *	0.2 *
5	0.3 *	0	0.2 *	−0.2 *	0.2 *	−0.1 *			0.1 *	0.1 *

**Table 4:** Results of the second electrical test after the post-mold cure process of the five test panels. Green stands for a calibrated signal and orange for a signal in a tolerance range. Gray marks the channels that were dysfunctional from the beginning, and red shows malfunctioning stacks after tempering. The * identifies a change and figures in bold type mark an improvement of the signal from the previous test.

Channel	Offset Panel 1		Offset Panel 2		Offset Panel 3		Offset Panel 4		Offset Panel 5	
	x	y	x	y	x	y	x	y	x	y
1	−0.8 *		−1.0 *	−0.1	0.3 *	−1.2	0 *	1.4 *	0.2 *	1.3 *
2	−0.8 *	1.5	−0.9 *	0.3	−1.1 *	−1.5 *	0.1	0.2 *	0.2	0.1 *
3	−1.0 *	1.5 *	0.2	0.1	0.2 *	0.1	0.1 *	0.1 *	0.2 *	1.5
4			0.4 *	0.2 *			−1.5 *	0	0.1 *	0 *
5	0.2 *	0	0.1 *	0.1 *	0.1 *	0 *			0.1	0.1 *

**Table 5:** Results of the fourth electrical test after the dicing process of test panels 2 and 5. Green stands for a calibrated signal and orange for a signal within the tolerance range. Gray marks the channels which were dysfunctional from the beginning and red shows malfunctioning stacks after dicing. The * identifies a change and figures in bold type mark an improvement of the signal from the previous test.

Channel	Offset Panel 2		Offset Panel 5	
	x	y	x	y
1	−1.3 *	0 *	0.2	1.5 *
2	−1.1 *	0.1 *	0.2	0.1
3	0.1 *	0.1	0.2	1.5
4	0.3 *	−0.1 *	0 *	0
5	0.1	0 *	0.1	0.1
